# Supplementary material for: Structural mapping of Nav1.7 antagonists
Source: Nat Commun. 2023 Jun 3;14:3224. doi: 10.1038/s41467-023-38942-3 (PMC10239435; doi:10.1038/s41467-023-38942-3)
Supplement: Supplementary file 1 — Supplementary Information [file 41467_2023_38942_MOESM1_ESM.pdf]

Supplementary information

Structural mapping of Na_V1.7 antagonists

Qiurong Wu[1,7], Jian Huang[2,7,8], Xiao Fan[2,7,8], Kan Wang[3,7], Xueqin Jin[1,7], Gaoxingyu Huang[4,5], Jiaao Li[1], Xiaojing Pan[1,8], and Nieng Yan[1,2,6,8]

[1]Beijing Frontier Research Center for Biological Structures, Tsinghua-Peking Joint Center for Life Sciences, School of Life Sciences, Tsinghua University, Beijing 100084, China

[2]Department of Molecular Biology, Princeton University, Princeton, NJ 08544, USA

[3]Department of Anesthesiology, China-Japan Friendship Hospital, Beijing 100029, China

[4]Westlake Laboratory of Life Sciences and Biomedicine, Key Laboratory of Structural Biology of Zhejiang Province, School of Life Sciences, Westlake University, 18 Shilongshan Road, Hangzhou 310024, Zhejiang Province, China

[5]Institute of Biology, Westlake Institute for Advanced Study, 18 Shilongshan Road, Hangzhou 310024, Zhejiang Province, China

[6]Shenzhen Medical Academy of Research and Translation, Guangming District, Shenzhen 518107, Guangdong Province, China

[7]These authors contributed equally to this work.

[8]To whom correspondence should be addressed: Nieng Yan (nyan@tsinghua.edu.cn); Xiaojing Pan (panxj@tsinghua.edu.cn); Jian Huang (jh6493@princeton.edu); Xiao Fan (xiaof@princeton.edu).

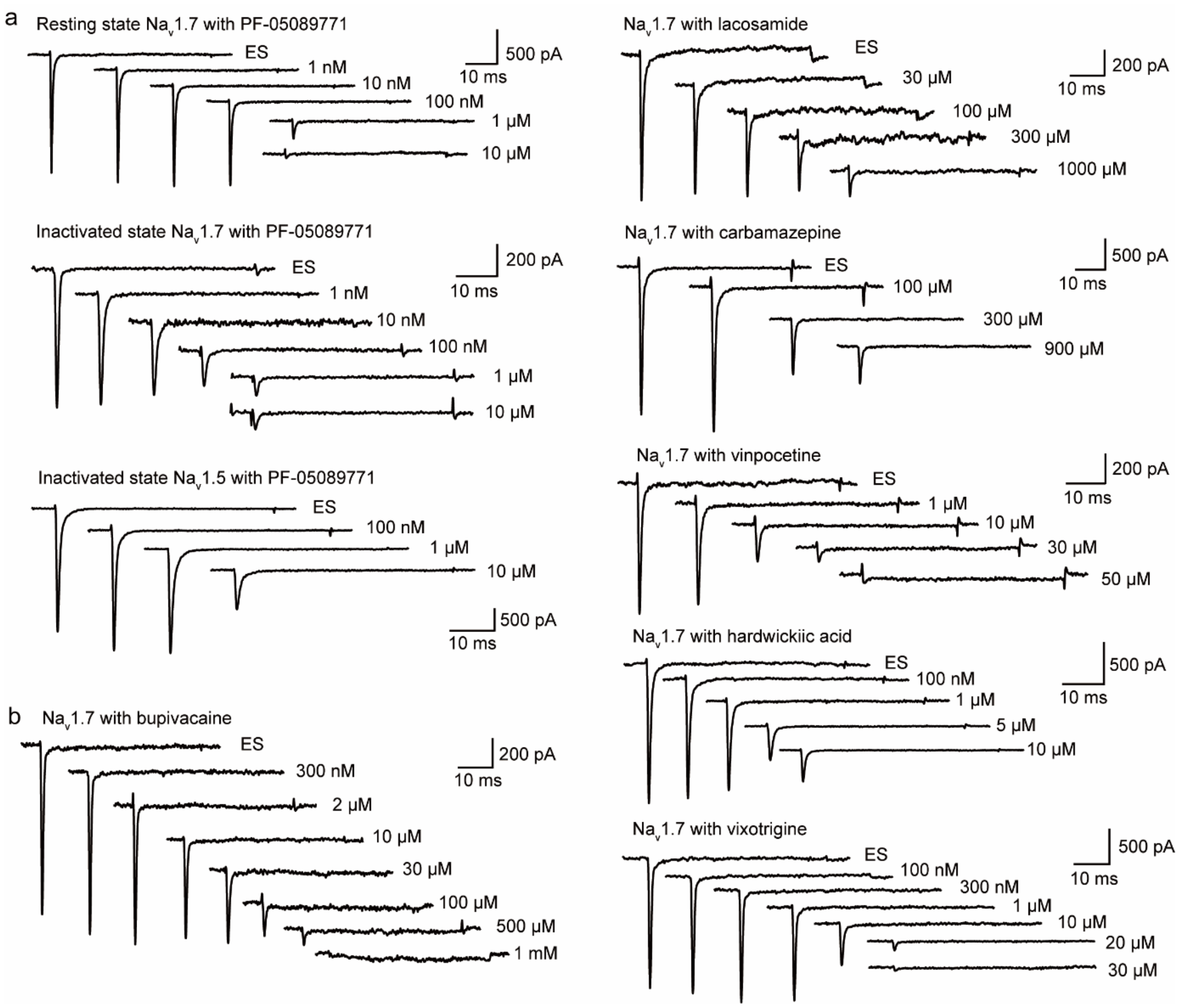

**Supplementary Fig. 1 | Blockage of Na$_v$1.7 by PF-05089771 and indicated drugs. a,** Representative traces for blocking Na$_v$1.7 and Na$_v$1.5 by PF-05089771 at indicated concentrations with resting or inactivated protocols. **b,** Representative traces for blocking of Na$_v$1.7 by bupivacaine, lacosamide, carbamazepine, vinpocetine, hardwickiic acid, and vixotrigine at indicated concentrations. Experimental details are presented in Supplementary Table 1 and Methods.

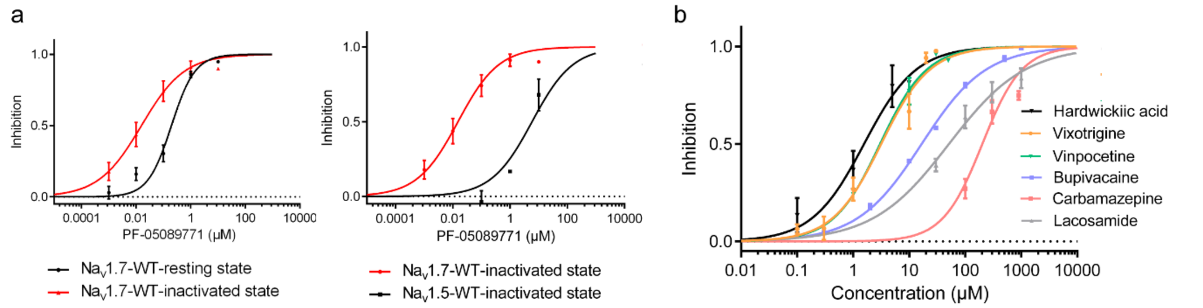

Supplementary Fig. 2 | Electrophysiological characterization of Na_v1.7 inhibition by PF-05089771 and various drugs. **a,** PF-05089771 specifically inhibits Na_v1.7. *Left*: Inhibition of Na_v1.7 by PF-05089771 with different recording protocols. Peak currents of Na_v1.7 with PF-05089771 applied at different concentrations were recorded with resting and inactivated protocols. *Right*: PF-05089771 is a Na_v1.7 subtype-selective inhibitor. Selectivity was assessed between Na_v1.5 and Na_v1.7 at their unique half-inactivation voltage for each channel. Na_v1.7-WT resting state, *n* = 4, 5, 8, 11,7. Na_v1.7-WT inactivated state, *n* = 10, 7, 6, 5, 2. Na_v1.5-WT inactivated state, *n* = 2, 2, 2. **b,** Inhibition of Na_v1.7 by indicated drugs. Peak currents of Na_v1.7 treated with indicated drugs applied at different concentrations were recorded with the resting protocols. Bupivacaine, *n* = 5, 5, 5, 5, 5, 5, 4. Lacosamine, *n* = 4, 4, 4, 4. Carbamazepine, *n* = 5, 5, 2. Vinpocetine, *n* = 3, 3, 1, 1. Hardwickiic acid, *n* = 1, 3, 2, 1. Vixotrigine, *n* = 3, 3, 3, 3, 2, 3. Data represent mean ± SEM. *n* biological independent cells. Experimental details are presented in Supplementary Table 1 and Methods.

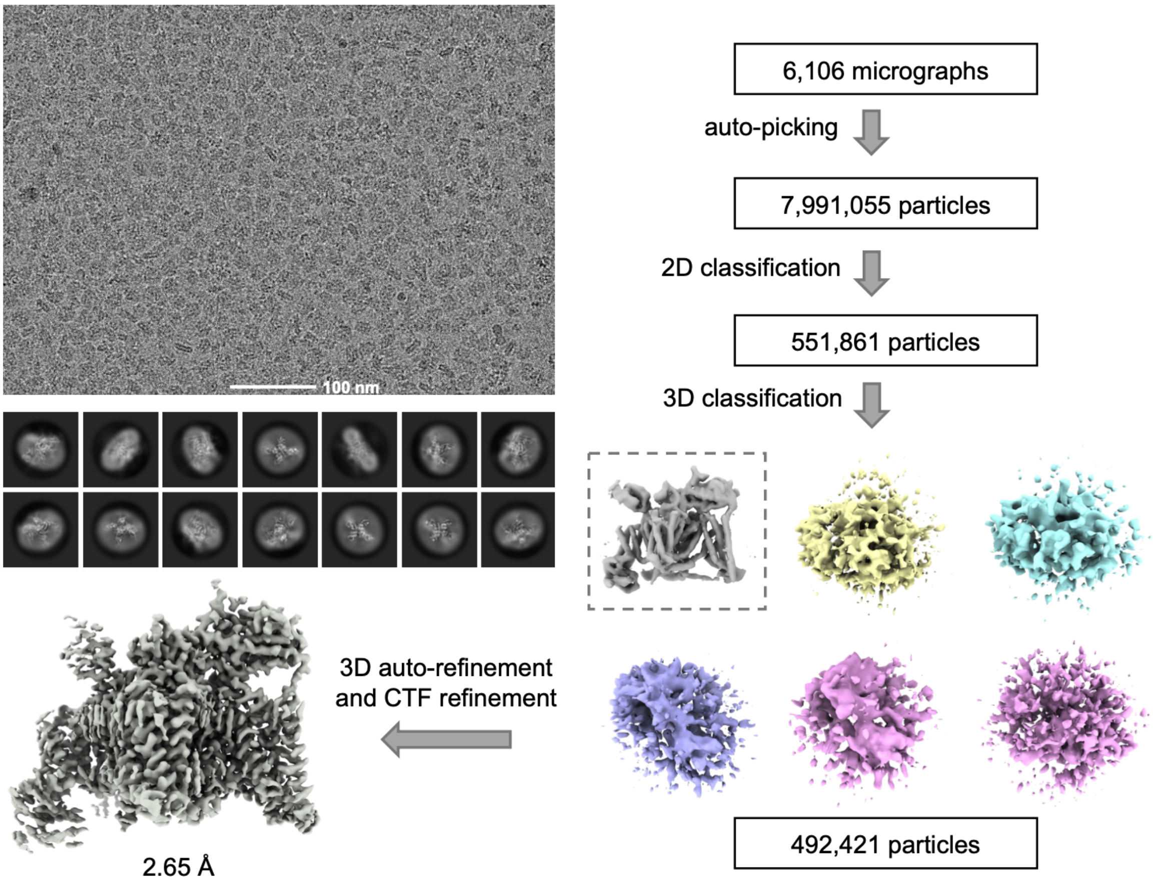

Supplementary Fig. 3 | Flowchart for EM data processing of Na$_v$1.7-β1-β2 treated with PF-05089771. Details can be found in Materials and Methods. Other datasets were processed following a similar protocol.

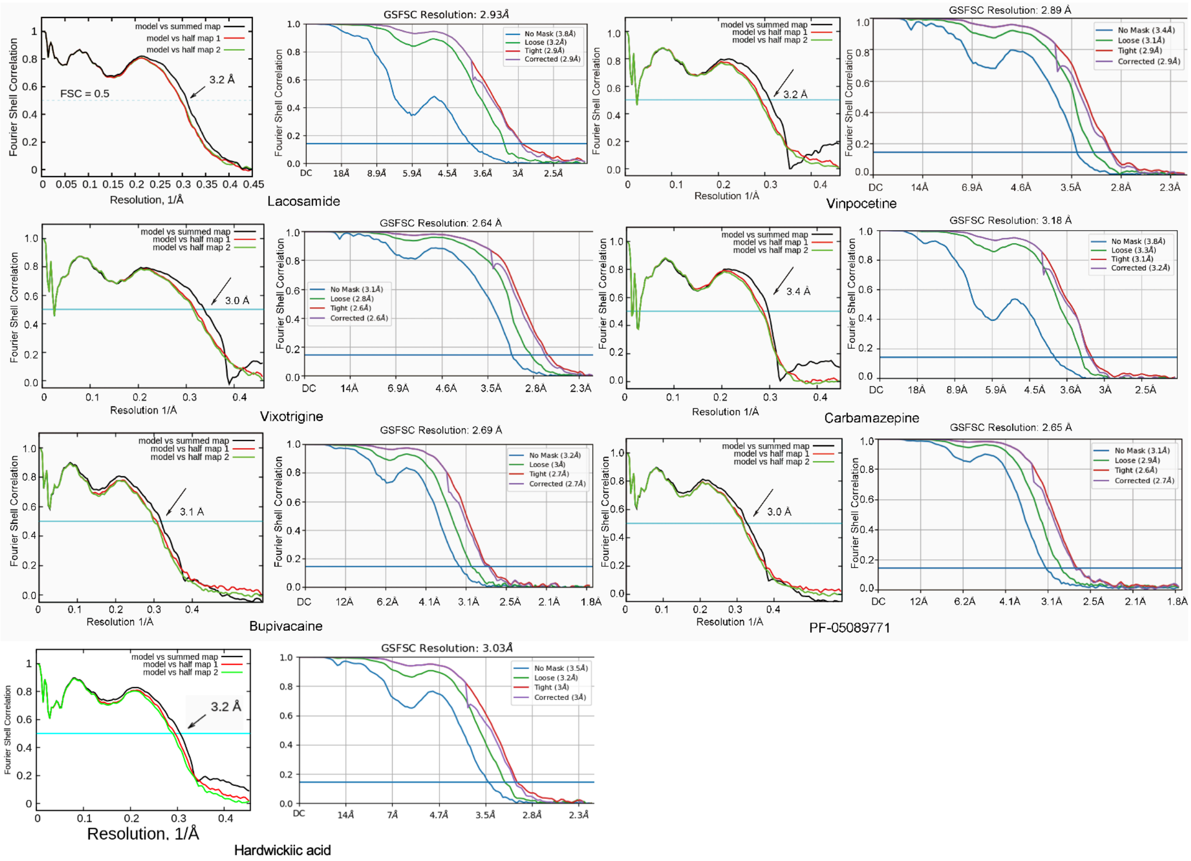

Supplementary Fig. 4 | Cryo-EM analysis of human Na$_V$1.7 with different drugs. For each indicated drug, left: FSC curves of the refined models against overall maps (black), of the respective model refined in the first of the two independent maps used for the gold standard FSC versus the same map (red), and of the model refined in the first of the two independent maps versus the second independent map (green). The small difference between the red and green curves indicates that the refinement did not suffer from overfitting. right: Gold standard FSC curves for the 3D reconstructions calculated in cryoSPARC.

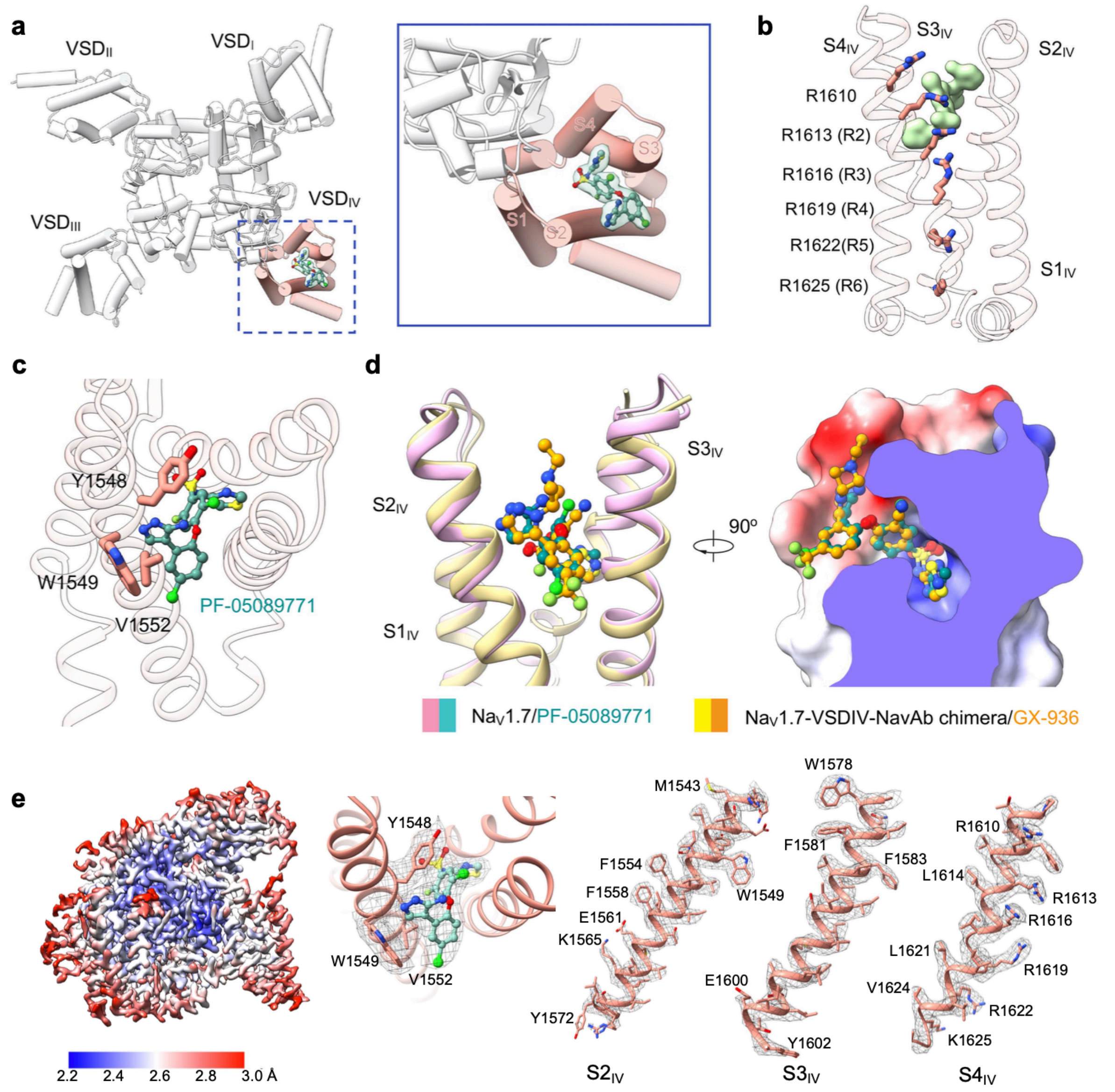

Supplementary Fig. 5 | PF-05089771 binds to Na$_v$1.7 VSD$_{IV}$. **a,** PF-05089771 resides in an extracellular cavity in Na$_v$1.7-VSD$_{IV}$. An extracellular view is shown with a close-up view in the inset. The density of PF-05089771, shown as green ball-and-sticks, is contoured in ChimeraX and presented as semi-transparent surface colored pale green. **b,** PF-05089771 inserts deeply in the VSD$_{IV}$ pocket. The gating charge residues on S4$_{IV}$ are shown as sticks. **c,** Coordination of PF-05089771. The ligand is shown as green ball-and- sticks, and the coordinating residues are shown as salmon sticks. **d,** The binding pose of PF-05089771 in Na$_v$1.7-VSD$_{IV}$ is nearly identical to that in Na$_v$1.7-VSD$_{IV}$-Na$_v$Ab chimera/GX-936. The structure of Na$_v$1.7-VSD$_{IV}$-Na$_v$Ab chimera/GX-936 (PDB code: 5EK0) is superimposed with that of Na$_v$1.7-VSD$_{IV}$. (Right) A cut-open view of the electrostatic surface of Na$_v$1.7-VSD$_{IV}$

is shown to highlight the pocket for PF-05089771. **e,** *Left:* local resolution distribution of the final reconstruction for the Nav1.7-PF-05089771-complex, estimated by cryoSPARC. *Middle and right:* EM density map of representative helices and residues involved in PF-05089771 binding.

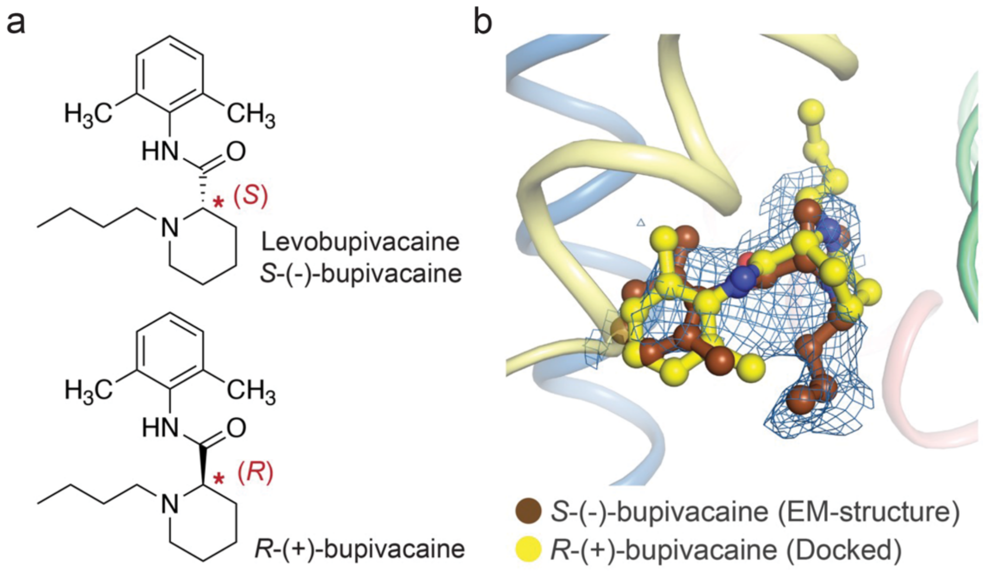

Supplementary Fig. 6 | Molecular docking simulation of bupivacaine isomers. a, Chemical structures of bupivacaine with different chirality. **b,** Molecular docking of bupivacaine isomers. Bupivacaine was docked against the structure of detergent-removed $Na_v1.7$ within Schrödinger Suite 2017-4 [1]. The structures of the protein and the compound were prepared by default setting using Protein Preparation Wizard and Ligprep program [2,3]. Molecular docking was subsequently performed using the Glide program with the extra-precision docking (Glide XP) method [4]. The top-scored pose of the ligand was chosen for further analysis.

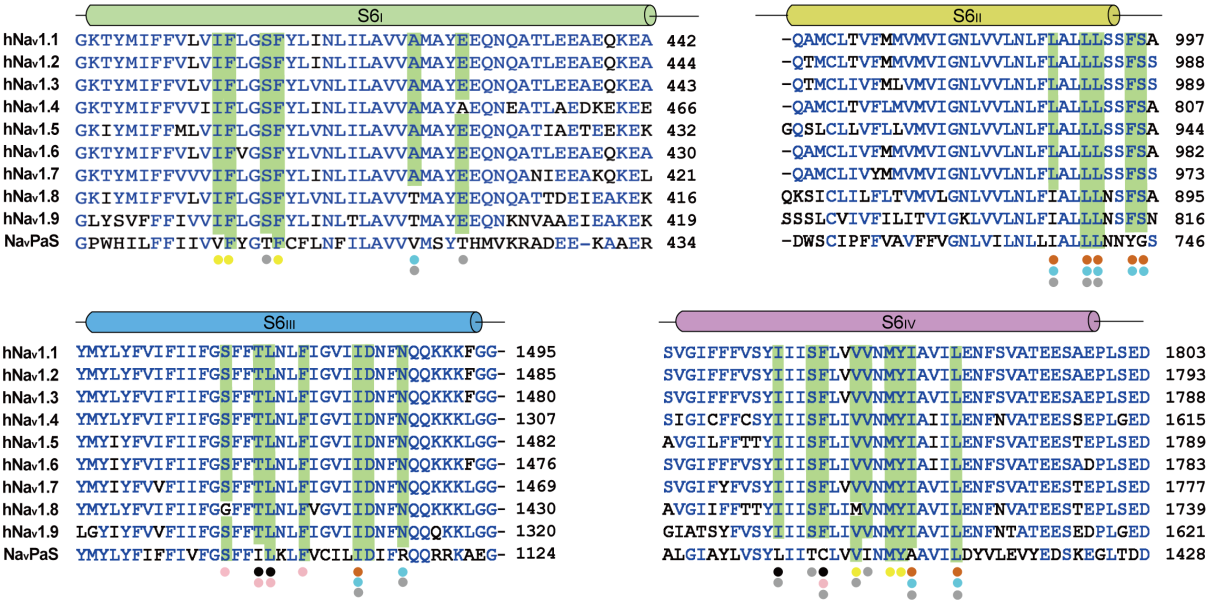

Supplementary Fig. 7 | Sequence alignment of the S6 segments in the four repeats of human Na$_v$ channels. Primary sequences are of the indicated Na$_v$ channels were aligned using Clustal Omega. Secondary structural elements are indicated above the sequences and color-coded for the four repeats. The residues involved in binding to bupivacaine (brown), lacosamide (grey), carbamazepine (cyan), vinpocetine (black), vixotrigine (yellow) and hardwickiic acid (pink) are indicated with spheres below the sequence alignment. The Uniprot IDs for the aligned human Na$_v$ sequences are: Na$_v$1.1: P35498; Na$_v$1.2: Q99250; Na$_v$1.3: Q9NY46; Na$_v$1.4: P35499; Na$_v$1.5: Q14524; Na$_v$1.6: Q9UQD0; Na$_v$1.7: Q15858; Na$_v$1.8: Q9Y5Y9; Na$_v$1.9: Q9UI33.

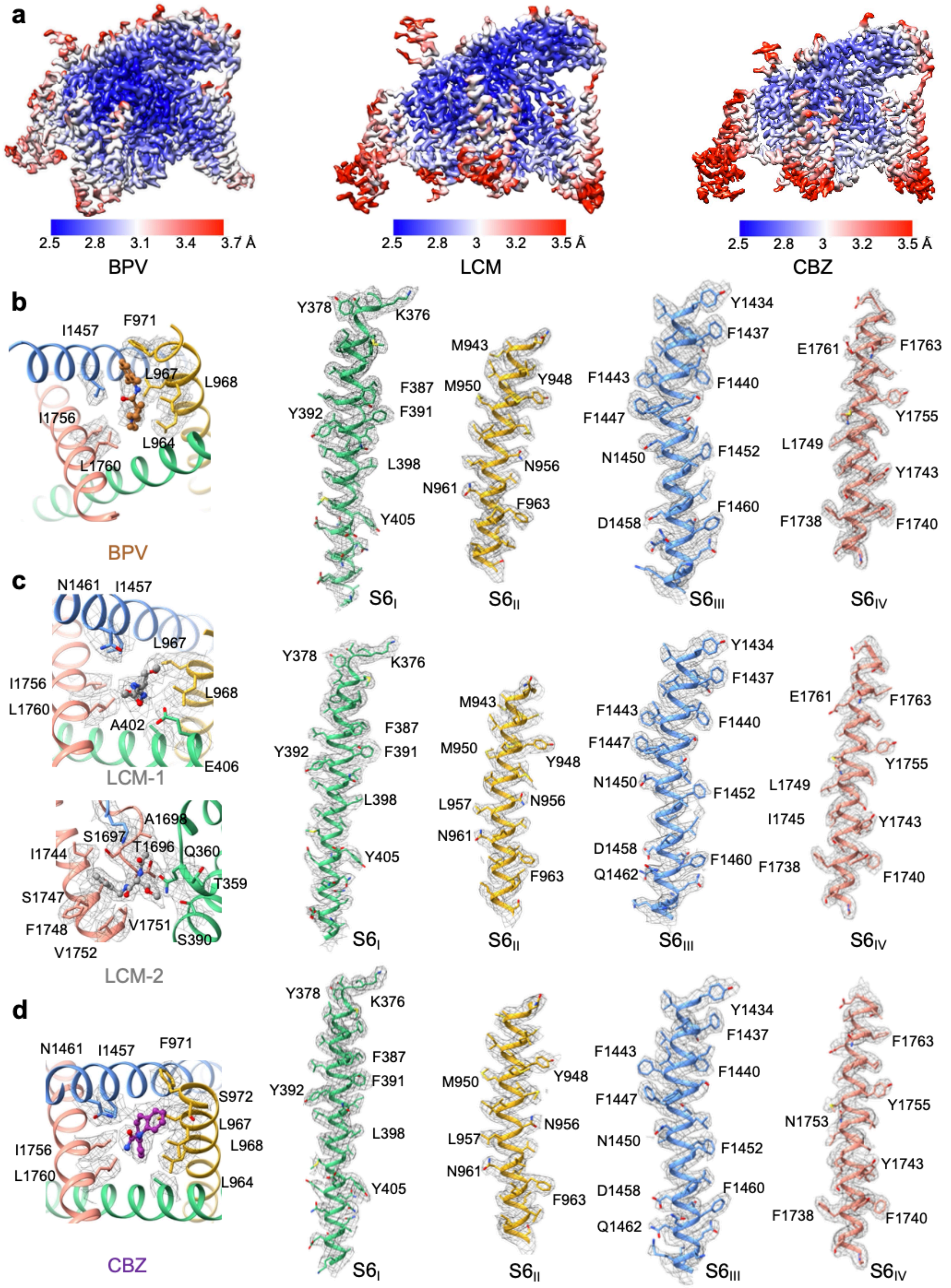

Supplementary Fig. 8 | Local resolution and EM density maps of representative segments involved in antagonists binding at site BIG. **a,** Local resolution distribution of the final reconstruction for Na_v1.7 in complex with BPV, LCM and CBZ, estimated by

cryoSPARC. **b,** Representative densities of the $Na_v1.7$-BPV complex. All densities shown here are contoured at 4 σ. **c,** Representative densities of $Na_v1.7$ in complex with LCM, and two binding sites densities are both exhibited. The densities of LCM-1 are contoured at 4 σ, while of LCM-2 are contoured at 5 σ. **d,** Representative EM densities of $Na_v1.7$ treated with CBZ. The densities shown here are contoured at 4.5 σ.

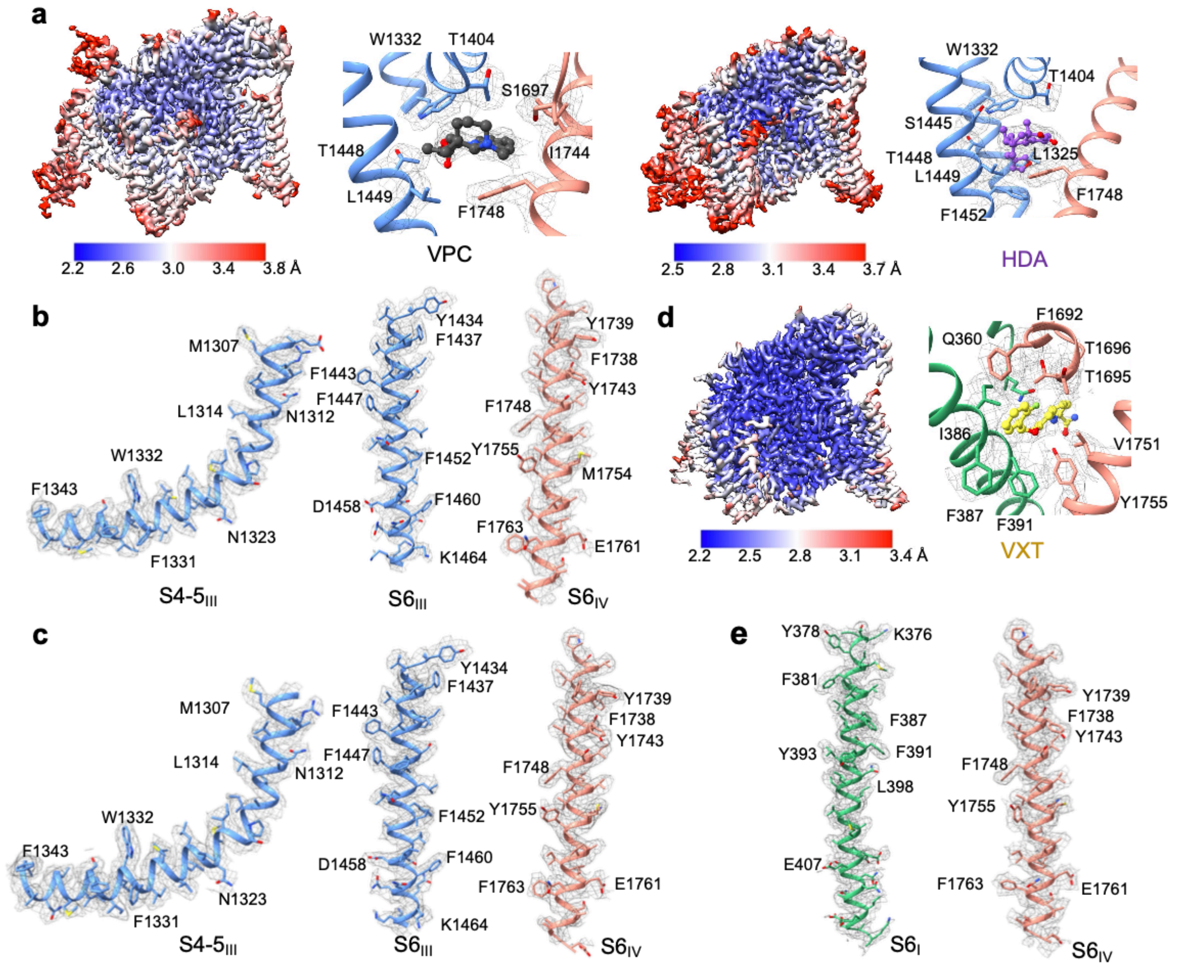

Supplementary Fig. 9 | Local resolution and EM density maps of representative segments that involved in antagonists binding at site F. **a,** Local resolution distribution and EM densities of the residues that involved in binding with VPC and HDA. The local resolution is estimated by cryoSPARC. **b,** Representative densities of the $Na_v1.7$-VPC complex. **c,** Representative densities of $Na_v1.7$ in complex with HDA. **d,** Local resolution and representative EM densities of $Na_v1.7$ treated with VXT. **e,** The EM density of representative segments which can interact with VXT.

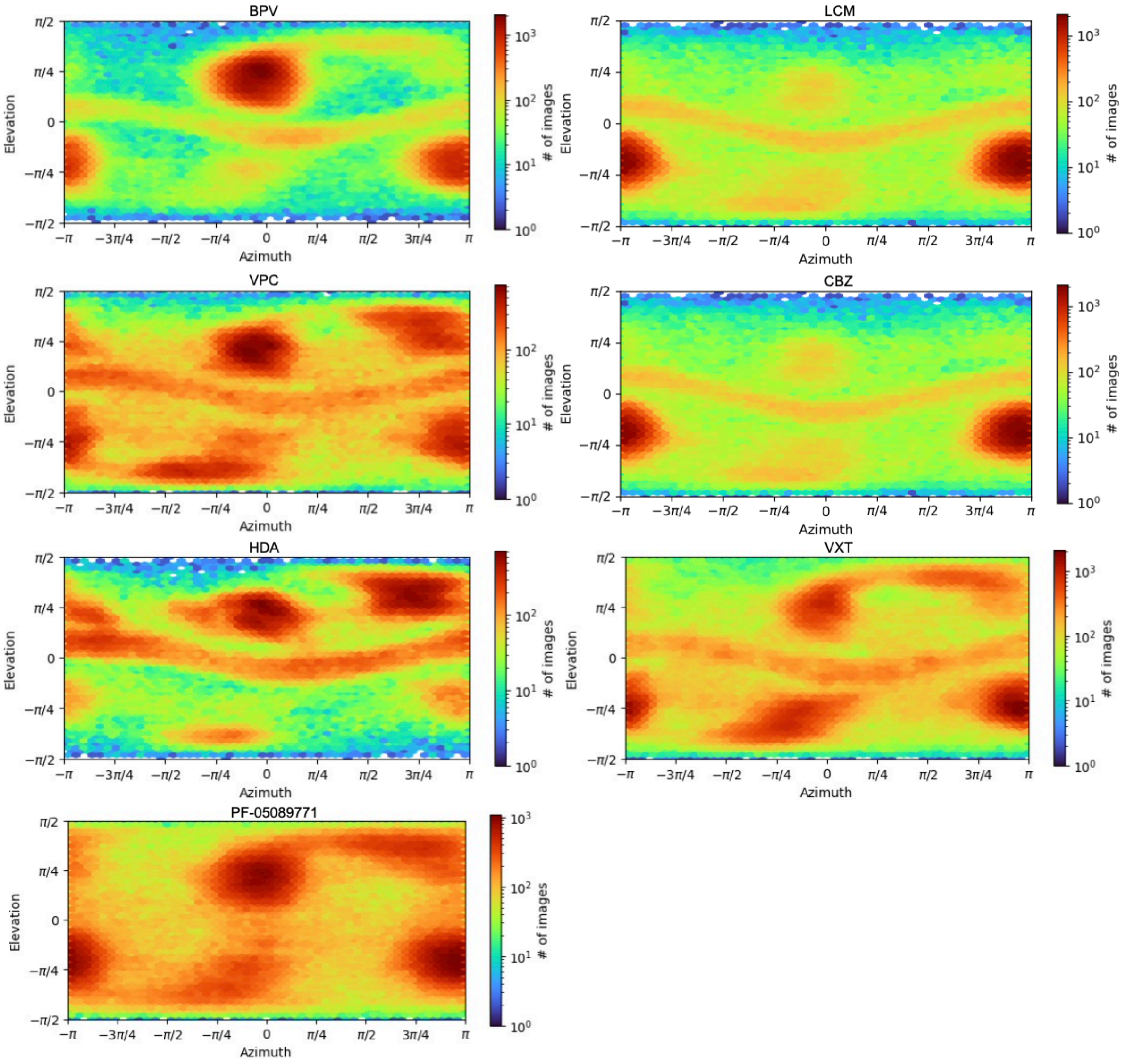

Supplementary Fig. 10 | Angular distribution of Na$_V$1.7 bound by different molecules.

Angular distribution of the final reconstruction of Na$_V$1.7 with seven different small molecules. All the figures are obtained from cryoSPARC.

Supplementary Table 1 | Concentration-response curves of PF-050897711 on Na_v1.7 and Na_v1.5 in HEK293T cells.

		PF-050897711 (Resting state, Na_v1.7)	PF-050897711 (Inactivated state, Na_v1.7)	PF-050897711 (Inactivated state, Na_v1.5)
IC_{50} (nM)		196.70 ± 24.80****	15.63 ± 3.96	4873 ± 1272.96****
P		< 0.0001	/	< 0.0001
Slope		1.01 ± 0.12*	0.55 ± 0.10	1.07 ± 0.26
P		0.0129	/	0.1402
n	1 nM	4	10	/
	10 nM	5	7	/
	100 nM	8	6	2
	1000 nM	11	5	2
	10000 nM	7	2	2

* P < 0.05 versus inactivated state Na_v1.7, **** P < 0.0001 versus inactivated state Na_v1.7. Each data point represents mean ± s.e.m (standard deviation of mean) and n is the number of experimental cells from which recordings were obtained. The extra sum-of-squares F test was used to compare the IC_{50} and slope factor of concentration-response curves. P values for IC_{50} comparatation: < 0.0001, Na_v1.7-WT resting sate v.s. Na_v1.7-WT inactivates sate; < 0.0001, Na_v1.5-WT inactivated sate v.s. Na_v1.7-WT inactivates sate. P values for slope computation: 0.0129, Na_v1.7-WT resting state v.s. Na_v1.7-WT inactivates sate; 0.1402, Na_v1.5-WT inactivated state v.s. Na_v1.7-WT inactivates sate.

Supplementary Table 2 | Concentration-response curves of different small molecule drugs on Na$_v$1.7.

<table>
<tr><th colspan="2"></th><th>BPV</th><th>LCM</th><th>CBZ</th><th>VPC</th><th>HDA</th><th>VXT</th></tr>
<tr><td colspan="2">IC_{50} (μM)</td><td>16.71 ± 0.63</td><td>51.31 ± 9.65</td><td>202.1 ± 28.21</td><td>3.03 ± 0.49</td><td>1.57 ± 0.40</td><td>3.16 ± 0.55</td></tr>
<tr><td colspan="2">Slope</td><td>0.76 ± 0.02</td><td>0.64 ± 0.11</td><td>1.23 ± 0.28</td><td>1.04 ± 0.13</td><td>0.93 ± 0.27</td><td>1.00 ± 0.13</td></tr>
<tr><td rowspan="14">n</td><td>100 nM</td><td>/</td><td>/</td><td rowspan="9">/</td><td>/</td><td>1</td><td>3</td></tr>
<tr><td>300 nM</td><td>5</td><td>/</td><td>/</td><td>/</td><td>3</td></tr>
<tr><td>1 μM</td><td>/</td><td>/</td><td>3</td><td>3</td><td>3</td></tr>
<tr><td>2 μM</td><td>5</td><td>/</td><td>/</td><td>/</td><td>/</td></tr>
<tr><td>5 μM</td><td>/</td><td>/</td><td>/</td><td>2</td><td>/</td></tr>
<tr><td>10 μM</td><td>5</td><td>/</td><td>3</td><td>1</td><td>3</td></tr>
<tr><td>20 μM</td><td>/</td><td>/</td><td>/</td><td rowspan="8">/</td><td>2</td></tr>
<tr><td>30 μM</td><td>5</td><td>4</td><td>1</td><td>3</td></tr>
<tr><td>50 μM</td><td>/</td><td>/</td><td>1</td><td rowspan="7">/</td></tr>
<tr><td>100 μM</td><td>5</td><td>4</td><td>5</td><td rowspan="5">/</td></tr>
<tr><td>300 μM</td><td>/</td><td>4</td><td>5</td></tr>
<tr><td>500 μM</td><td>5</td><td>/</td><td>/</td></tr>
<tr><td>900 μM</td><td>/</td><td>/</td><td>2</td></tr>
<tr><td>1 mM</td><td>4</td><td>4</td><td>/</td></tr>
</table>

Each data point represents mean ± s.e.m and n is the number of experimental cells from which recordings were obtained.

Supplementary Table 3 | Statistics for data collection and model refinement.

**Drugs**	**LCM**	**CBZ**	**BPV**	**PF**	**VXT**	**VPC**	**HDA**
**Data collection**							
EM equipment	FEI Titan Krios						
Voltage (kV)	300						
Detector	Gatan K2 Summit		Gatan K3 Summit				
Pixel size (Å)	1.114		0.865		1.0825		1.0979
Electron dose (e^-/Å^2)	50						
Defocus range	-1.4 ~ -1.2		-1.8 ~ -1.5				
Number of collected stacks	4,268	4,254	7,361	6,106	5,280	4,534	6,389
**Reconstruction**							
Software	RELION 3.1/cryoSPARC						
Symmetry	C1						
Initial particles	2,771,449	3,467,720	9,086,591	7,991,055	9,224,142	7,862,450	17,556,212
Final particles	300,001	287,917	297,950	492,421	535,763	304,324	220,477
Resolution (Å)	2.9	3.2	2.7	2.7	2.6	2.9	3.1
Map sharpening B-factor (Å^2)	-117.7	-128.9	-96.3	-104.3	-94.5	-98.1	-109.1
**Refinement**							
Software	Phenix						
Cell dimensions							
a=b=c (Å)	356.48	356.48	249.12	249.12	277.12	277.12	281.06
α=β=γ (˚)	90						
Model							
Non-hydrogen	13,409	13,534	13,448	13,839	13,640	13,590	12,419
Protein	1,565	1,565	1,564	1,705	1,565	1,565	1,448
Ligand	2	1	1	1	1	1	1
R.m.s deviations							
Bonds length	0.006	0.006	0.003	0.005	0.003	0.004	0.004
Bonds Angle	0.772	0.756	0.602	0.715	0.572	0.707	0.671
Ramachandran plot statistics (%)							
Preferred	94.45	94.84	96.51	96.68	98.32	98.19	98.04
Allowed	5.55	5.16	3.49	3.32	1.68	1.81	1.96
Outlier	0.00	0.00	0.00	0.00	0.06	0.00	0.00

Supplementary Table 5. Chemical Structures of NaV-binding small molecules

A. Toxins

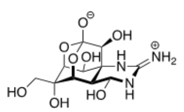	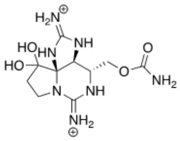	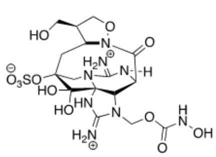
Tetrodotoxin (TTX)<br>(PDB: 6J8J, 6J8I, 7W9M)	Saxitoxin (STX)<br>(PDB: 6J8H, 6J8G, 7W9T, 7W9P)	Zetekitoxin AB (ZTX)

B. Clinical Drugs

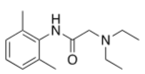	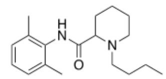	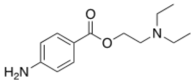	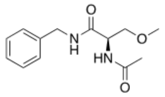
**Lidocaine**<br>**(No density)**	**Bupivacaine (BPV)**<br>**(This work)**	Procaine	**Lacosamide (LCM)**<br>**(This work)**
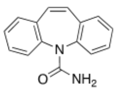	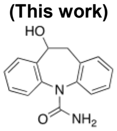	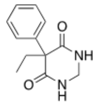	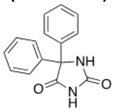
**Carbamazepine (CBZ)**<br>**(This work)**	Licarbazepine	Primidone	Phenytoin
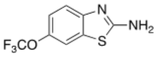	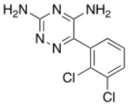	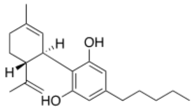	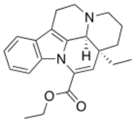
Riluzole	Lamotrigine	Cannabidiol<br>(PDB: 8G1A)	**Vinpocetine (VPC)**<br>**(This work)**

C. Molecules under development

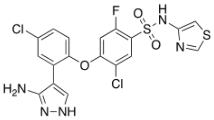	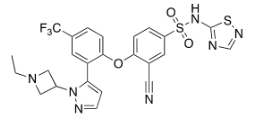	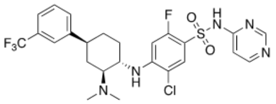	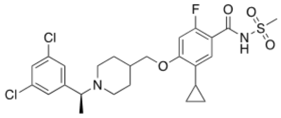
**PF-05089771**<br>**(This work)**	GX-936<br>(PDB: 5EK0, Na_v1.7-VSD4-Na_vAb)	GNE-3565<br>(PDB: 8F0R)	GDC-0310<br>(PDB: 8F0Q)
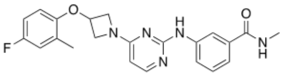	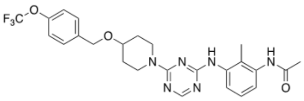	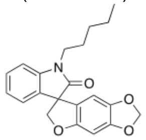	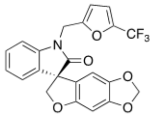
Nav1.7-IN-2<br>(PDB: 7XMF)	TC-N 1752<br>(PDB: 7XMG)	XEN907<br>(PDB: 7XM9)	**Funapide**<br>**(Limited resolution)**
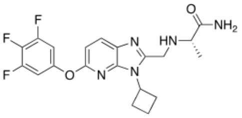	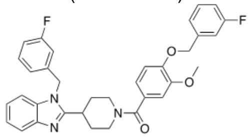	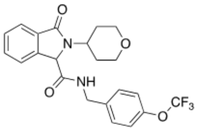	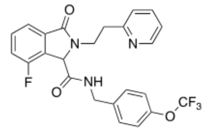
**DSP-2230**<br>**(Limited resolution)**	AZ194	NAV 26	Sodium Channel Inhibitor 1
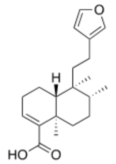	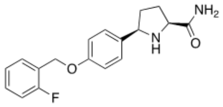	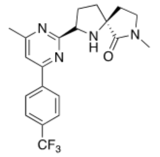	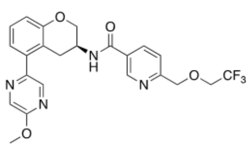
**Hardwickiic acid (HDA)**<br>**(This work)**	**Vixotrigine (VXT)**<br>**(This work)**	BIIB095	AZD-3161
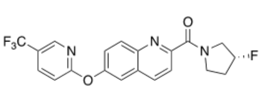	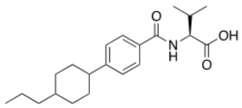	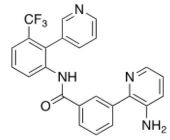	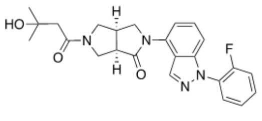
ABBV-318	GNE-0439	cmpd from Amgen	cmpd from Abbvie

Bold font is used to indicate the molecules tested in this work, including the small molecules that have resolved high-resolution structures and the ones we tried but found no density or corresponding resolution too poor to define.

Supplementary References:

1. *Schrödinger Release 2017-4*, (Schrödinger, LLC., 2017).
2. Sastry, G.M., Adzhigirey, M., Day, T., Annabhimoju, R. & Sherman, W. Protein and ligand preparation: parameters, protocols, and influence on virtual screening enrichments. *J Comput Aided Mol Des* **27**, 221-34 (2013).
3. Harder, E. et al. OPLS3: A Force Field Providing Broad Coverage of Drug-like Small Molecules and Proteins. *J Chem Theory Comput* **12**, 281-96 (2016).
4. Friesner, R.A. et al. Extra precision glide: docking and scoring incorporating a model of hydrophobic enclosure for protein-ligand complexes. *J Med Chem* **49**, 6177-96 (2006).
